# Combining Viedma Ripening and Temperature Cycling Deracemization

**DOI:** 10.1021/acs.cgd.1c01423

**Published:** 2022-01-31

**Authors:** Giuseppe Belletti, Jelle Schuurman, Hester Stinesen, Hugo Meekes, Floris P. J. T. Rutjes, Elias Vlieg

**Affiliations:** Institute for Molecules and Materials, Radboud University, Heyendaalseweg 135, 6525 AJ Nijmegen, The Netherlands

## Abstract

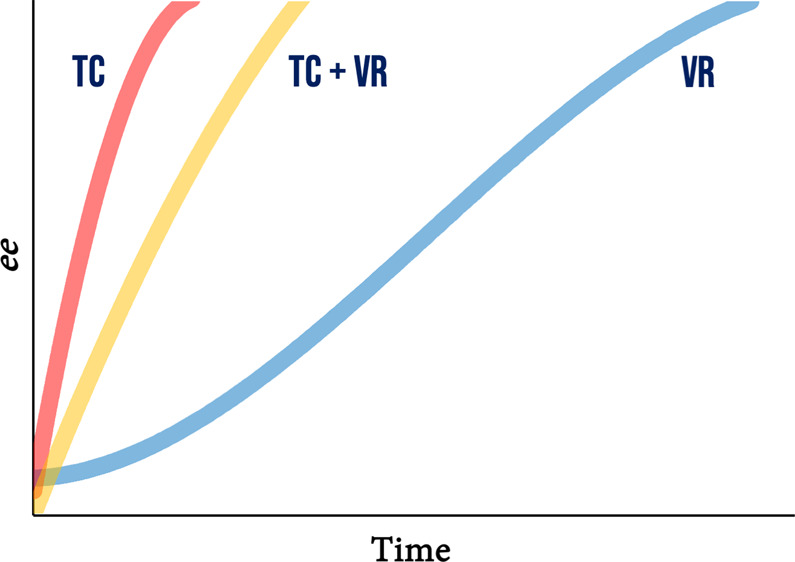

While much data are available for the Viedma ripening and temperature cycling deracemization processes, not much is known about the advantages (or disadvantages) of a combination of the two. We here try to elucidate what happens when Viedma ripening is used in combination with temperature cycling by comparing not only the deracemization times but also the change in the sizes of the crystals. We found that, in the case of NMPA (*rac*-(2-methylbenzylidene)-phenylglycine amide) as a model compound, combined experiments significantly increase the deracemization time. By tuning the process parameters, it is possible to approach experimental conditions where both Viedma ripening and temperature cycling control the deracemization. Under those conditions, however, the deracemization time is not significantly improved. Following our results, it seems unlikely that a combination of Viedma ripening and temperature cycling would shorten the deracemization time. Nevertheless, these experiments might provide clues for unraveling the mechanism of temperature cycling.

## Abstract 2

For deracemization, temperature cycling (TC) is in general faster than Viedma ripening (VR). The combination of the two methods leads to intermediate deracemization times.

## Introduction

Viedma ripening and temperature cycling have emerged in the past decade as very robust and reliable deracemization techniques.^1−8^ Because they both employ the solid state of chiral compounds, one of the requirements for both processes is that the two enantiomers crystallize in separate crystals, i.e., as a racemic conglomerate. (A very recent paper by Viedma and Ortiz suggests that for a combination of Viedma ripening and temperature fluctuations, even racemic compounds may be deracemized.^9^) The other prerequisite, which further narrows the applicability of the two techniques, is that the model compounds racemize in solution: in this way the two enantiomers continuously interconvert into each other and the chiral amplification of the enantiomeric excess (*ee*) in the solids can take place.^10^ Despite these limitations, both processes have been proven successful for a wide number of compounds, including pharmaceuticals, coordination complexes and organometallic compounds.^4,11−18^ In several examples in which both Viedma ripening and temperature cycling were applied to the same compound, the latter technique was shown to proceed with a higher rate.^19^ It was also evident that both processes display an exponential trend,^19−22^ suggesting in both cases a self-amplification mechanism which has already been explained for the Viedma ripening process.^10^ Although exhibiting similar curves, it is still not clear how to explain the rather large difference in the two deracemization rates.

While the Viedma ripening mechanism is currently well accepted,^10,23^ for temperature cycling, a few mechanisms have been proposed by different groups.^24−27^ None of them, however, fully explain the existing experimental results and thus leave the subject open for further interpretation. Aside from understanding the behavior behind temperature cycling, to our knowledge, the combination of the two techniques was never applied in a single experiment. The question that we aim to clarify here is whether combining Viedma ripening and temperature cycling would shorten the deracemization time and what the differences are with respect to the separate use of the two processes. Combined experiments could also provide additional experimental data to develop consistent models that could describe both processes.

In our work, we use *rac*-(2-methylbenzylidene)-phenylglycine amide (NMPA) as a model compound, a Schiff-base derivative of the amino acid phenylglycine and the first organic compound to be deracemized through Viedma ripening.^2^ Over the course of the past years, NMPA was used for many studies with either Viedma ripening or temperature cycling and, for this reason, it represents a reliable compound for which much information is available.^2,19,22,28,29^ Noorduin et al. reported the successful deracemization of NMPA in their first attempt to apply Viedma ripening to an organic molecule.^2^ One decade later, Breveglieri et al. deracemized the same compound using temperature cycling.^22^ The completion times were significantly different and were dependent on the initial *ee*. For Viedma ripening, an initial *ee* as high as 10% would provide complete deracemization in at least 5 days. In the case of temperature cycling, a similar starting *ee* guarantees a completion time of 24 h or less. Of course other parameters such as the amount of compound, the amount of racemization catalyst, and the grinding speed in the case of Viedma ripening or the temperature difference (Δ*T*) in temperature cycling can influence the deracemization time.

In this work, we find that the addition of grinding to temperature cycling experiments increases the deracemization time. This effect is larger for higher grinding speeds and is pronounced for a Δ*T* where temperature cycling can be considered the dominating mechanism. For smaller Δ*T*, grinding has a stronger influence on the deracemization time. In this case, the system is approaching the Viedma ripening regime. As a consequence, for small values of Δ*T*, combined experiments show a faster completion time than a pure temperature cycling experiment, but the overall deracemization time is still much longer than that for larger Δ*T*. Measurement of the crystal sizes shows that the average size of the crystals reduces considerably when grinding is introduced.

## Experimental Section

The model compound used in this study, NMPA, was synthesized following the reported procedure.^2^ All the experiments were performed in 20 mL glass vials which were sealed and positioned inside a double-jacketed vessel, connected to the thermostat with which the temperature was controlled. The temperature profiles chosen consisted of two constant temperatures separating a cooling and a heating ramp. The lower temperature was always kept at 22 °C, whereas the higher temperature varied according to the different Δ*T* values used. The cycles were repeated continuously until deracemization was completed. In all the experiments, 0.72 g of *rac*-NMPA and 0.08 g of (*R*)-NMPA were mixed together and homogenized in 10 mL of methanol for about 30 min, before 80 μL of racemization catalyst DBU was added. In this way, all the experiments started with an initial *ee* of approximately 10%. The time zero of each experiment corresponded to the time the DBU was added to the suspension. For the pure temperature cycling experiments, no glass beads were used but only gently stirring at 300 rpm. During the combined experiments, a fixed amount of glass beads (3 g, 2 mm diameter) and a stirring bar were used. Samples were taken at the lowest temperature by extracting about 40 μL of suspension using a 20–200 μL micropipet, vacuum filtered, and washed with ethanol, in which the compound has a very poor solubility. The *ee* was measured using an Agilent chiral HPLC instrument equipped with a Chirobiotic T column, a 0.5 mL/min flow, and methanol as the eluent. The retention times were 7.5 and 8.0 min for the (*S*)- and the (*R*)-enantiomer, respectively. Scanning electron microscopy (SEM) images of the crystals were analyzed with a Phenom scanning electron microscope, using a magnification of 750×.

## Results

**Figure 1:**
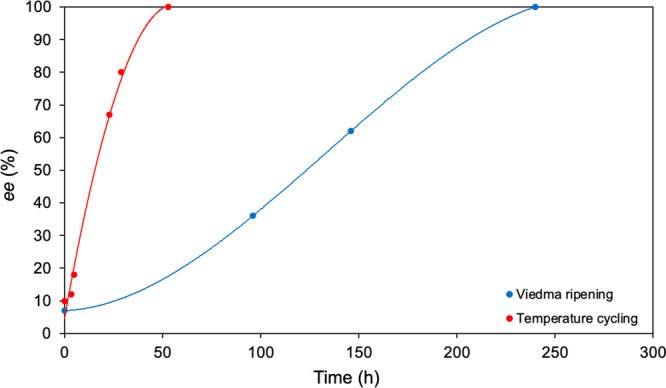
Viedma ripening (blue curve) and temperature cycling (red curve) deracemization of NMPA with an initial *ee* of approximately 10%. The lines are a guide to the eye.

In order to have a reference time for the combined experiments we first deracemized NMPA using both Viedma ripening and temperature cycling separately. The experimental conditions were as similar as possible for both techniques, i.e., same initial *ee* of approximately 10%, same amount of material, same amount of DBU, and same solvent concentration. In accordance with the results of Noorduin et al.^2^ and Breveglieri et al.,^22^Figure 1 shows that the completion times of the two techniques differ significantly; i.e., temperature cycling is approximately 5 times faster than Viedma ripening for the chosen reference conditions. The Viedma ripening experiment was performed using a grinding speed of 1000 rpm, whereas the cycle used in temperature cycling consisted of two constant temperatures of 22 and 40 °C (Δ*T* = 18 °C) held for 20 min, a heating ramp of 30 min, and a cooling ramp of 50 min, so a total cycle length of 2 h.

**Figure 2:**
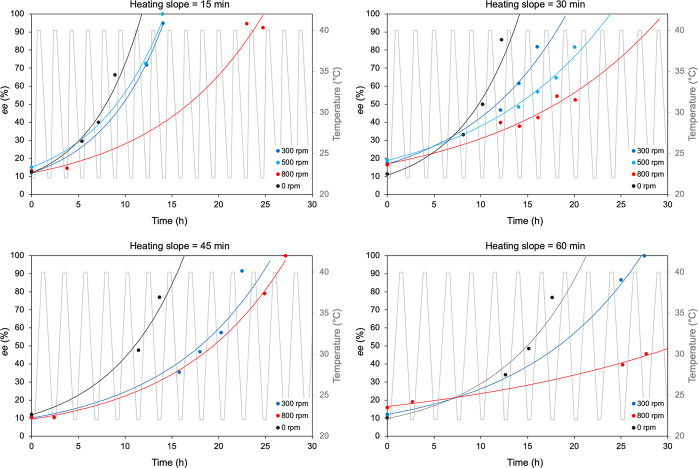
Overview of the combination experiments using grinding speeds of 300, 500, and 800 rpm with heating slopes of 15, 30, 45, and 60 min for temperature cycles with Δ*T* = 18 °C (22–40 °C). For each heating slope, a temperature cycling experiment is shown as a reference. This is indicated with 0 rpm, meaning that no glass beads were used and only a stirring speed of 300 rpm was used. The lines are a guide to the eye.

Experiments on the combination of Viedma ripening and temperature cycling were performed using two different approaches: (i) adding the grinding to a regular temperature cycling experiment with Δ*T* = 18 °C (22–40 °C); (ii) reducing Δ*T* to approach the Viedma ripening experimental conditions. In addition, the change in the size of the crystals was monitored for all of the experiments, by means of SEM. The effect of the grinding on a temperature cycling experiment with a Δ*T* = 18 °C was studied by applying different grinding speeds, namely, 300, 500, and 800 rpm. An equal amount of glass beads was used as the grinding media in all of the experiments. The other experimental conditions such as the amount of material, DBU and solvent were kept constant in all of the experiments. As a reference, a temperature cycling experiment was performed with no glass beads at a 300 rpm stirring rate. Furthermore, to compare the results for different temperature profiles, experiments with an equal Δ*T* but various heating times were performed. In Figure 2, the results can be found for heating times of 15, 30, 45, and 60 min. In this way, we could compare not only different grinding speeds but also temperature cycles with a different heating rate that used the same grinding speed. Note that 0 rpm means that no glass beads were present during the deracemization but that only a gentle stirring speed of 300 rpm was applied. The results are shown in Figure 2, grouped by the different heating rates investigated. Unfortunately, experiments using a grinding speed of 500 rpm were not successful for the heating slopes of 45 and 60 min. We therefore decided not to include them in the study. It is evident that, regardless of the heating slope, the deracemization time increases with the grinding speed and that the temperature cycling experiments are always faster than the combined experiments. In addition, the overall deracemization time slightly increases when increasing the heating time. These results seem to indicate that for Δ*T* = 18 °C, regardless of the heating rate, Viedma ripening slows down a regular temperature cycling experiment.

**Figure 3:**
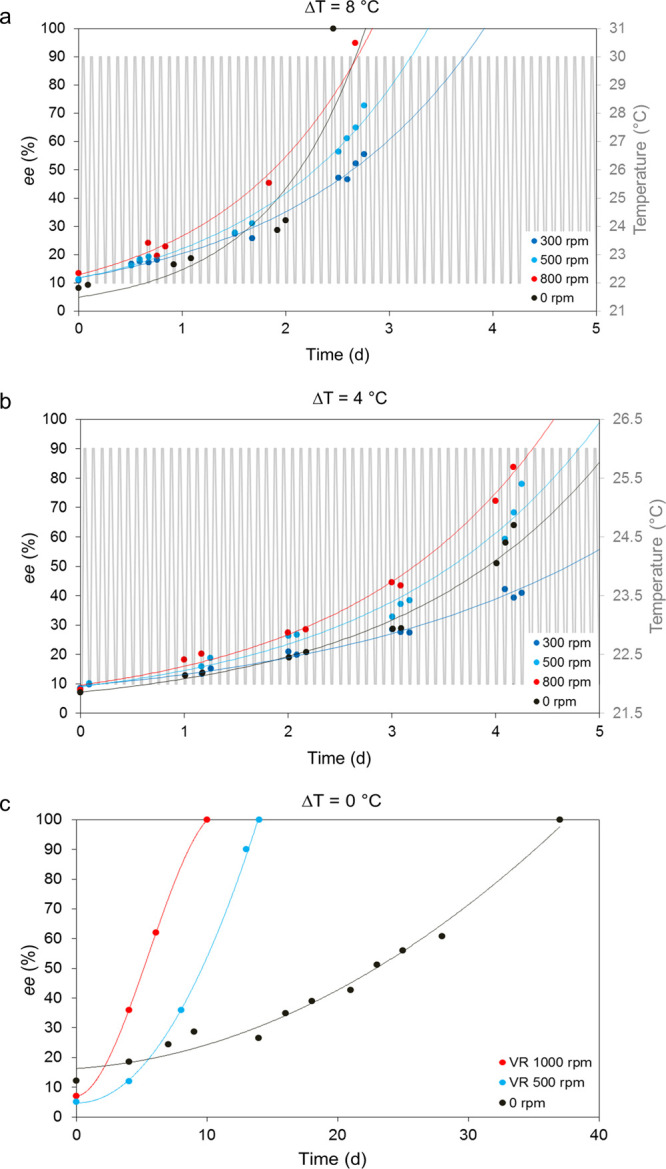
Overview of the combination experiments using (a) Δ*T* = 8 °C (22–30 °C); (b) Δ*T* = 4 °C (22–26 °C); (c) Δ*T* = 0 °C (22 °C). In (a) and (b), grinding speeds of 300, 500, and 800 rpm were used. Note that only a heating slope of 30 min was tested. The curves indicated with 0 rpm correspond to temperature cycling experiments where no glass beads were used and only a stirring speed of 300 rpm was used. The lines are a guide to the eye.

Combined experiments that used temperature cycles of different (and lower) Δ*T* values were also performed. Like for the previous set of experiments, most of the parameters were kept constant, i.e., the amount of material, DBU, solvent, and glass beads and the heating ramp (30 min). The Δ*T* values tested were 8, 4, and 0 °C. In all cases, the lower temperature was kept at 22 °C. Reducing the Δ*T* allows an approach to the regime of Viedma ripening, where grinding is expected to have the most significant impact on the deracemization. The results are shown in Figure 3.

These experiments show an opposite trend compared to the experiments with a larger Δ*T* of 18 °C. The overall deracemization time is longer, and grinding has now a substantial influence on the combined experiments. Specifically, the higher the attrition rate, the faster the process reaches enantiopurity. This effect is more pronounced for smaller Δ*T*. Figure 3b shows that combined experiments with 500 and 800 rpm grinding speed are even faster than a pure temperature cycling experiment. A possible reason for this is that, by reducing Δ*T*, the system approaches the Viedma ripening regime, where the boost given by the attrition to the combined experiments is stronger than for larger Δ*T*. The limiting case, namely Δ*T* = 0 °C, Figure 3c, shows that without grinding complete deracemization takes about 35 days. When grinding is applied, the deracemization time increases with a decrease in the attrition rate. This situation is comparable to a Viedma ripening experiment for which grinding is an essential feature of the mechanism.^10^ To clearly see the trends in the experiments just discussed, we summarized the results of Figures 2 and 3 in Tables 1 and 2, respectively.

**Table 1:** Deracemization Time (in h) of the Combined Experiments Using Four Grinding Speeds and Four Heating Slopes for Temperature Cycles with Δ*T* = 18 °C (22–40 °C)^a^

	grinding speed (rpm)			
heating rate (min)	0	300	500	800
15	12	14	14	25
30	14	19	24	29
45	17	25		27
60	21	27		50

a For each heating slope, a temperature cycling experiment (indicated with 0 rpm) is shown as a reference. Experiments that used a grinding speed of 500 rpm failed for the heating slopes of 45 and 60 min and are therefore not included.

**Table 2:** Deracemization Time (in h) of the Combined Experiments Using Four Grinding Speeds and Four Δ*T* Values^a^

	grinding speed (rpm)			
Δ*T* (°C)	0	300	500	800
18	14	19	24	29
8	59	95	82	68
4	130	160	122	110
0	910		335	240^b^

a In all of the experiments, the heating ramp time was of 30 min and the lower temperature was kept at 22 °C. For each Δ*T*, a temperature cycling experiment (indicated with 0 rpm) is shown as a reference. No experiment was done for the combination of Δ*T* = 0 and 300 rpm grinding speed.

b 1000 rpm.

**Figure 4:**
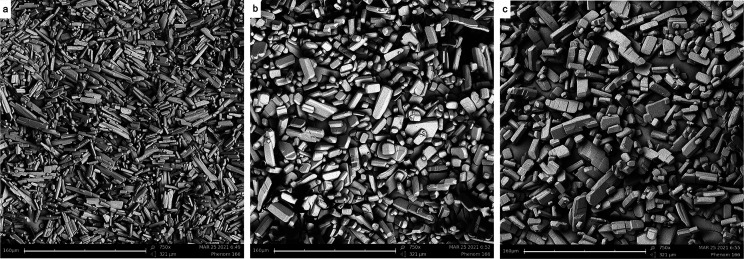
SEM images taken during a typical temperature cycling experiment representative of NMPA (a) at the start of the deracemization, roughly 18% *ee*; (b) at 50% *ee*, and (c) at the end of the deracemization. The scale bar at the bottom of each picture corresponds to a length of 160 μm. The average crystal size is (a) 20 μm, (b) 30 μm, and (c) 32 μm. All the images have the same magnification of 750×.

To investigate the effect of the combined experiments on the crystal size, SEM images of the crystals after completion of the deracemization were taken and analyzed. SEM images taken during a pure temperature cycling deracemization experiment are also reported, Figure 4. These images correspond to values of the *ee* of 18, 50, and 100%. We see that the average crystal size increases up to a value of 50% *ee* but after that remains relatively constant until the end of the deracemization. This finding was already suggested by Suwannasang et al. in one of the first temperature cycling experiments on a chiral substrate.^8^

**Figure 5:**
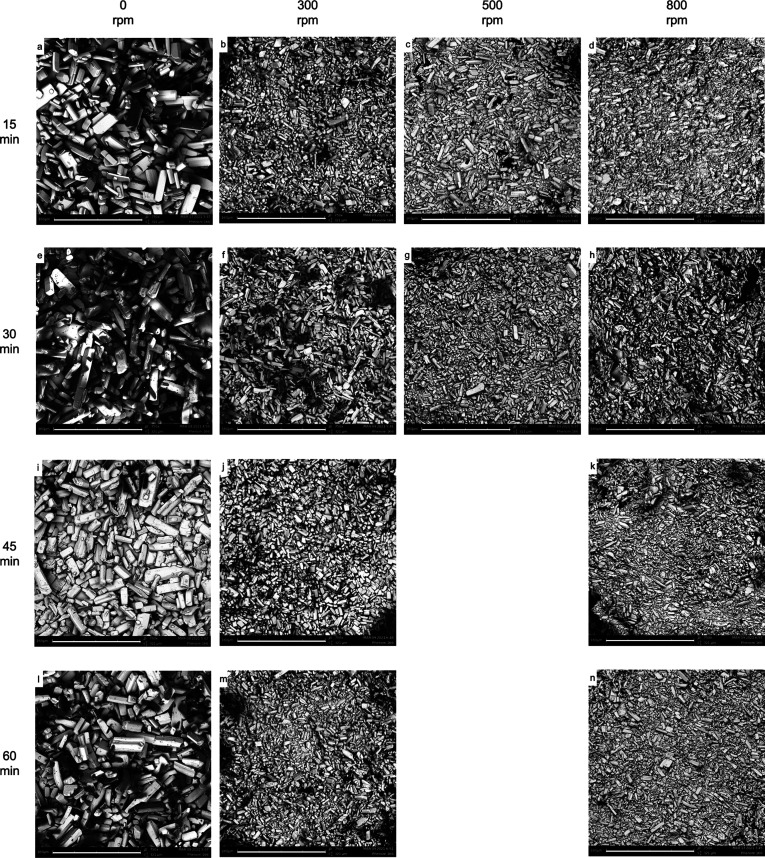
SEM images of the crystals at the end of the deracemization of the combined experiments with a Δ*T* of 18 °C. Images of the crystals at 0 rpm (no grinding but gentle stirring speed) correspond to pure temperature cycling experiments. Images of the stirring rate of 500 rpm for the 45 and 60 min heating slopes are missing due to unsuccessful experiments. The scale bar at the bottom of each picture corresponds to a length of 160 μm. All the images have the same magnification of 750×.

SEM images of the combined experiments discussed in Figure 2, namely when Δ*T* = 18 °C, are shown in Figure 5. For each set, a picture of the crystals after a mere temperature cycling experiment (0 rpm grinding speed) is shown as a reference. Clear trends are observable for all the different heating times: an increase in the grinding speed corresponds to a reduction in the size of the crystals. In fact, in a typical Viedma ripening process the breakage rate of the crystals is enhanced with the grinding speed.^2^ Therefore, one would expect as a result a high number of smaller crystals for high attrition rates. Although for a Δ*T* of 18 °C the system is mostly governed by temperature cycling, the images show that the effect of the grinding is still pronounced and cannot be neglected. Conversely, experiments using only temperature cycling, and no grinding, display the biggest sizes of NMPA crystals.

A summary of all the average sizes for each combination of grinding speed/heating rate is reported in Table 3. These refer only to experiments that used a Δ*T* of 18 °C. Together with these values, the sizes of the crystals after temperature cycling experiments (0 rpm grinding speed) are also shown as a reference.

**Table 3:** Average Crystal Sizes (in μm) of NMPA Crystals after Temperature Cycling Experiments (No Grinding, 0 rpm) and Combined Viedma Ripening and Temperature Cycling Experiments for the Different Heating Slope/Grinding Speed Combinations^a^

	grinding speed (rpm)			
heating rate (min)	0	300	500	800
15	29.3	11.5	13.5	9.1
30	35.5	14.2	12.5	9.4
45	33.2	11.7		9.2
60	36.7	12.1		8.6

a Experiments that used a grinding speed of 500 rpm failed for the heating slopes of 45 and 60 min and are therefore not included.

**Figure 6:**
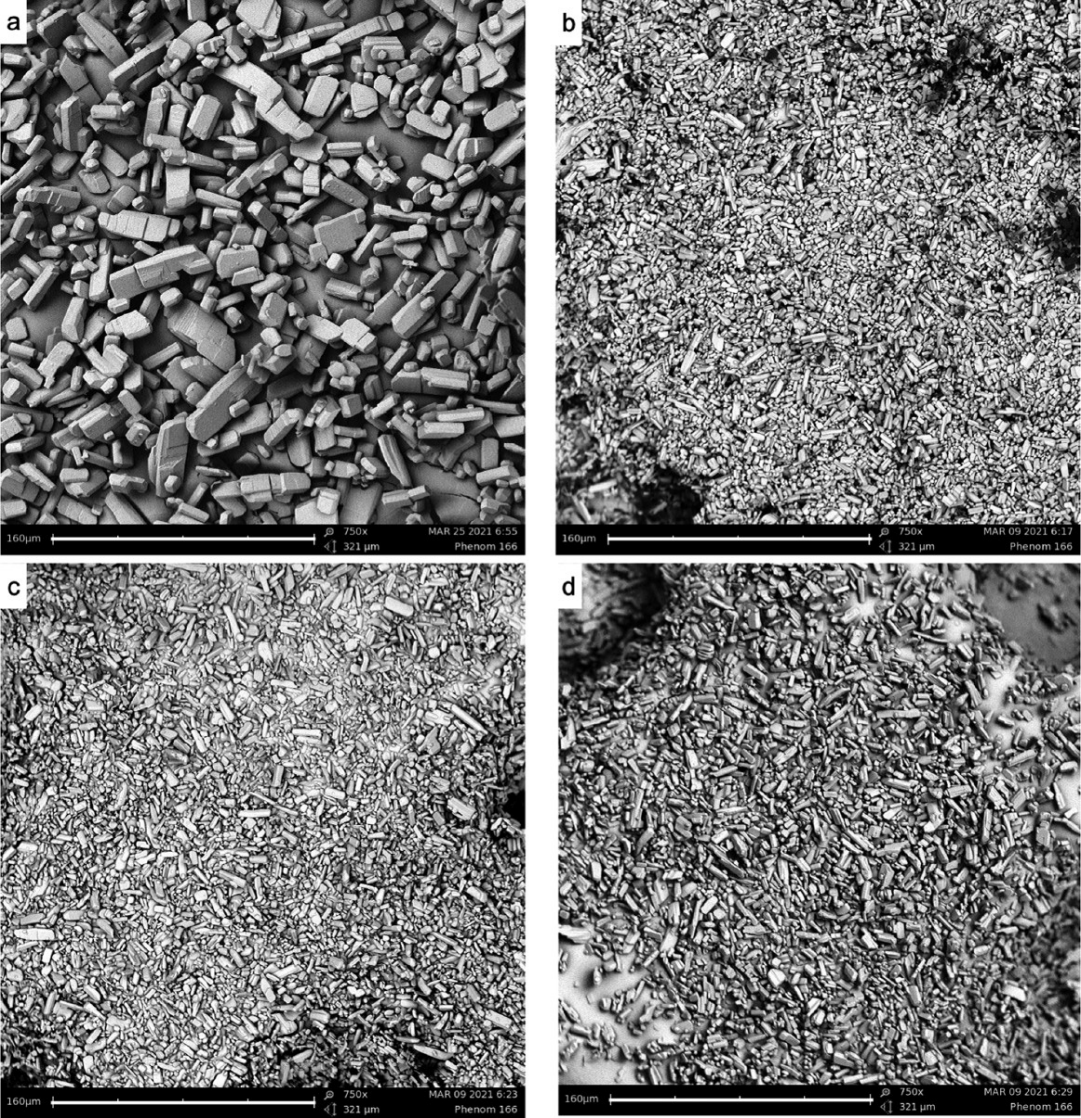
SEM images taken at the end of the combined experiments using a Δ*T* of 8 °C (22–30 °C) showing NMPA crystals at grinding speeds of (b) 300 rpm, (c) 500 rpm, and (d) 800 rpm. (a) Crystals of a temperature cycling experiment with Δ*T* = 8 °C and a gentle stirring speed of 300 rpm, as a reference. The scale bar at the bottom of each picture corresponds to a length of 160 μm. All the images have the same magnification of 750×.

**Figure 7:**
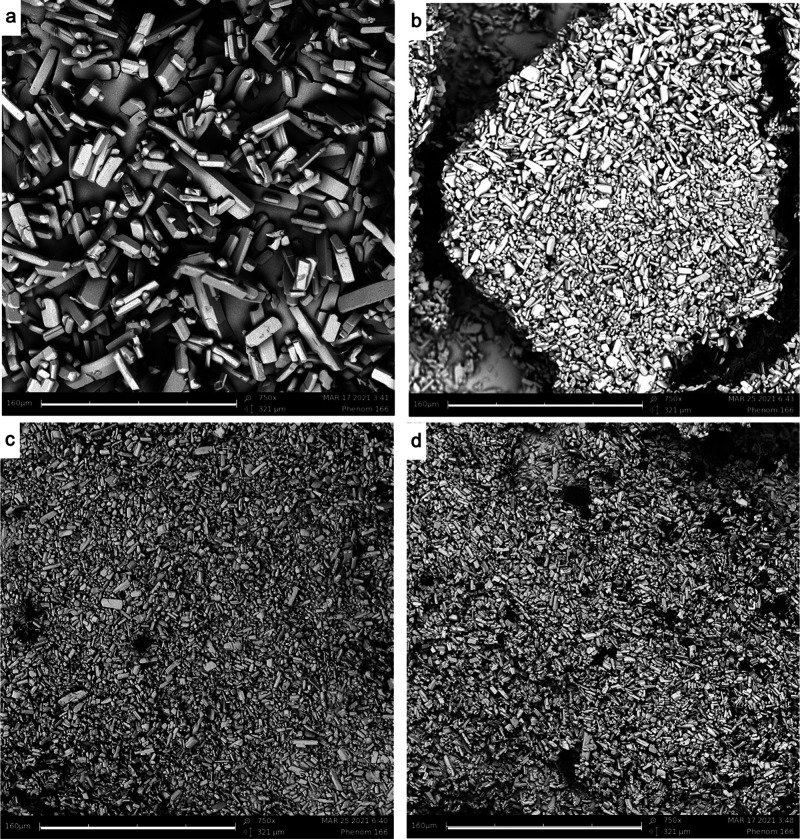
SEM images taken at the end of the combined experiments using a Δ*T* of 4 °C (22–26 °C) showing NMPA crystals at grinding speeds of (b) 300 rpm, (c) 500 rpm, and (d) 800 rpm. (a) Crystals of a temperature cycling experiment with Δ*T* = 4 °C and a gentle stirring speed of 300 rpm, as a reference. The scale bar at the bottom of each picture corresponds to a length of 160 μm. All the images have the same magnification of 750×.

SEM images of the combined experiments with a smaller Δ*T*, namely, Δ*T* = 8 °C and Δ*T* = 4 °C, were also analyzed. These are reported in Figures 6 and 7, respectively. The top left images of Figures 6 and 7 display the NMPA crystals after a temperature cycling experiment. It is evident that, in both sets of pictures, the overall crystal size is smaller than for the combined experiments with a larger Δ*T* of 18 °C.

## Discussion

Figure 2 shows two important trends. When only temperature cycling experiments are taken into consideration, these reach enantiopurity faster than any combined experiments. In addition, the completion rate seems to be related to the heating slope: the shorter the heating step (or dissolution), the faster the deracemization. The second important information displayed in Figure 2 is that when grinding is applied to the system, this evolves to homochirality slower. Different grinding speeds result in different completion times: the higher the attrition, the longer the time to reach enantiopurity. This observation indicates that the effect of the grinding is to reduce the deracemization efficiency in experimental conditions that mostly favor temperature cycling as the dominating process, i.e. for large Δ*T*. SEM images in Figure 5 suggest that this reduction is related to the decrease in crystal size during the experiment.

Important information is also obtained from Figure 3, i.e., where Δ*T* is smaller. When compared to the experiments performed with a Δ*T* of 18 °C, only in the case of Δ*T* = 8 °C did temperature cycling yield homochirality (slightly) faster than any combined experiment. It is here evident that the grinding has a considerable effect on the system: the overall deracemization time is now longer than that for experiments with a larger Δ*T*; however, this effect decreases with an increase in the attrition rate. For the combined experiments that used a Δ*T* of 4 °C, Figure 3b, the effect is even more pronounced: fast attrition rates proceed to enantiopurity faster than low attrition rates, with the difference being larger than that for a larger Δ*T*, Figure 3a. Moreover, in this case, attrition rates equal to or higher than 500 rpm show a faster completion time than that for temperature cycling only (Figure 3b). This reveals a sort of inversion of the trend with respect to the combined experiments performed at Δ*T* = 18 °C. In fact, grinding has now such a significant influence on the system that it appears to become the dominating mechanism. SEM images in Figures 6 and 7 show that the crystal sizes of the different attrition rates are now generally smaller when compared to a larger temperature difference. As can be seen in Figure 3, the smaller the Δ*T*, the longer the deracemization time. From the Viedma ripening perspective, however, it can be said that the introduction of even a small Δ*T* to the system leads to a much faster deracemization time. This is displayed in Table 2: when moving from Δ*T* = 0 °C to Δ*T* = 4 °C, shorter completion times can be observed, regardless of the grinding speed.

All of the above observations hold for chiral compounds but might differ in the case of achiral compounds. To date, two examples reported in literature show that, for achiral molecules that crystallize in a chiral space group, such as sodium chlorate (NaClO_3_) and sodium bromate (NaBrO_3_), temperature cycling was found to be slower than Viedma ripening.^30,31^ Additionally, in the case of sodium bromate, it was shown that vigorous grinding of the crystals during a temperature cycling experiment resulted in much shorter completion times.^30^ This raises the question of whether there are other situations where Viedma ripening is preferable over temperature cycling. For certain molecules, there could be limitations on the experimental conditions. An example could be a compound with such a high solubility that a significant Δ*T* is not possible. In these cases, Viedma ripening might result in a better (and more reliable) deracemization process. As our system shows, however, even then a small Δ*T* may still decrease the total deracemization time.

For this chiral system, our results indicate that when temperature cycling is the dominating mechanism, i.e., for large temperature differences, the addition of the grinding leads to longer deracemization times. In this scenario, pure temperature cycling experiments are always proven to be the fastest. Reducing the Δ*T* of the temperature cycle allows an approach to conditions where Viedma ripening has a strong impact on the deracemization time and takes control over the process. However, the overall deracemization rate is considerably lowered. Therefore, combined experiments seem to not be beneficial for the deracemization of NMPA. This could be different in the case of other substrates, either chiral or achiral. In this case, (very) small temperature differences could potentially help speed up the process.

## Conclusions

We have shown that, for NMPA, when grinding is added to a regular temperature cycling experiment, the completion time of the deracemization increases with the grinding rate. This holds for experimental conditions for which temperature cycling is dominating, namely, for large temperature differences. In this situation, pure temperature cycling experiments always proceed to homochirality with the fastest rate. As a parallel effect, the average crystal size turns out to decrease with the increase of the attrition rate. When Δ*T* is decreased, the overall deracemization time increases. In this case, the attrition can have a large effect on the combined experiments. For small enough Δ*T* values, an increase in the grinding speed shortens the deracemization time with respect to the pure temperature cycling experiments that use the same Δ*T*. This is also true for the limiting case, Δ*T* = 0 °C, which is a normal Viedma ripening experiment. These results show that the outcome of a combined temperature cycling/Viedma ripening experiment depends on the process conditions. For NMPA, pure temperature cycling experiments with Δ*T* of 18 °C give the fastest results and the combined experiments seem to not be beneficial. This might differ for compounds for which experimental conditions are restricted and Viedma ripening could be the preferential deracemization technique. In this situation, the introduction of small temperature differences could help speed up the process. The results presented here provide additional data toward a more consistent understanding of both Viedma ripening and temperature cycling.
